# Turning resistance into therapeutic vulnerability: Mechano-reprogramming in cancer

**DOI:** 10.1016/j.mbm.2026.100209

**Published:** 2026-08-22

**Authors:** Yunjia Qu, Yingxiao Wang

**Affiliations:** a Alfred E. Mann Department of Biomedical Engineering, Department of Quantitative Computational Biology, Department of Stem Cell & Regenerative Medicine, Department of Molecular Microbiology & Immunology, University of Southern California, Los Angeles, CA, 90089, USA; b Norris Comprehensive Cancer Center, University of Southern California, Los Angeles, CA, 90033, USA

## Abstract

Cancer mechanics is often framed as a progression from compliant normal tissue to a stiff, fibrotic tumor. Yet this tissue-level description conceals a second and therapeutically consequential feature: rigid tumors can contain mechanically soft cancer-cell states and localized compliant niches. These mechanically soft states are associated with stem-like plasticity, invasive potential and resistance to cytotoxic lymphocytes. Converting the mechanical signals that define these resistant states into therapeutic outputs may open a new direction for solid-tumor immunotherapy.

## Graphical abstract

**Figure:**
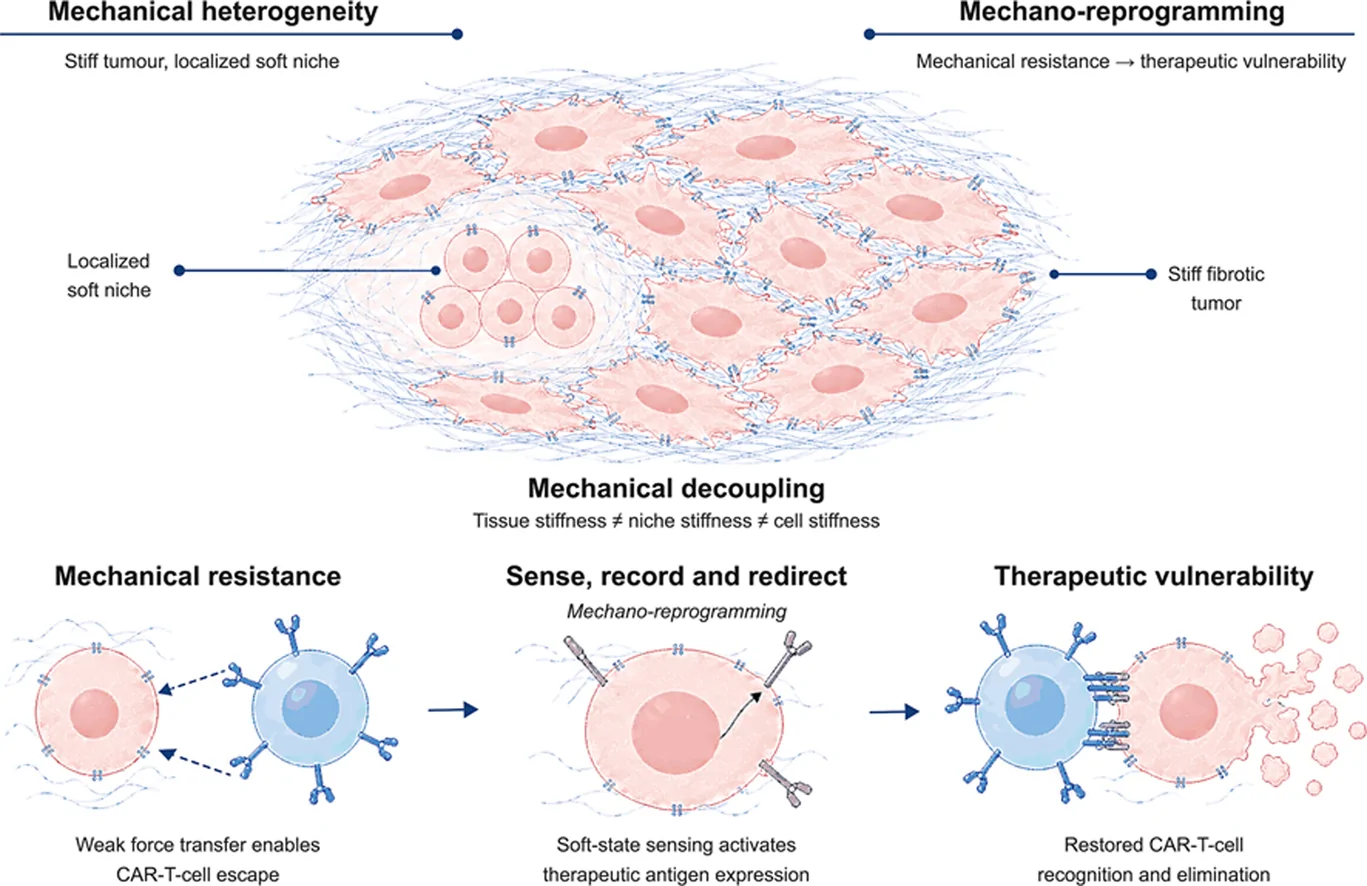

Physical properties of tumors are active regulators of malignant behavior rather than passive consequences of disease. Fibrosis and collagen deposition increase the rigidity of many solid tumors and disturb normal tissue tensional homeostasis,^1^ while matrix crosslinking further promotes tumor progression by enhancing integrin signaling.^2^ This stiff extracellular-matrix (ECM) framework has been foundational because it connects tissue architecture to proliferation, invasion and treatment failure. However, tissue-level stiffness does not describe every mechanical state within a tumor. A fibrotic tumor can contain localized compliant regions and mechanically soft cancer cells. These localized compliant regions may form soft niches that harbor rare cancer-cell states associated with relapse and immune escape.

Mechanical measurements across scales support this view. Tumor-associated fibrosis alters ECM architecture, cellular organization, fluid pressure and solid stress, which collectively shape bulk-tissue mechanics. Cellular stiffness, by contrast, reflects the plasma membrane, cortex, cytoskeleton, nucleus and active state of an individual cell. Tissue stiffness and cell stiffness therefore describe related but distinct properties. Atomic-force-microscopy mapping of intact breast specimens revealed relatively uniform stiffness in normal and benign tissues but broad mechanical distributions in malignant tissues, including a prominent low-stiffness population.^3^ Measurements in solid tumors later showed directly that rigid tumors contain soft cancer cells.^4^ Thus, soft regions and soft cancer cells are not merely structural features of mechanically heterogeneous tumors; they may mark the microenvironments and cell states most capable of invasion and disease progression. Experimental models provide a functional basis for this association. Soft three-dimensional fibrin matrices enrich tumor-repopulating cells with strong self-renewal and tumor-forming capacity,^5^ while matrix softness further maintains this plasticity through H3K9 demethylation and Sox2 expression.^6^ Cancer cells can also create soft compliant paths through collagen-rich tissue by MMP14-dependent local matrix remodeling, thereby facilitating invasion.^7^ We therefore use the term “soft niche” to describe a localized soft or compliant microenvironment that selects, maintains or enables malignant cell states within a globally stiff tumor. Such niches may arise from pre-existing variations in ECM organization or be generated dynamically as invasive cells remodel their surroundings. Importantly, not every region with a low measured modulus necessarily constitutes a functional niche. Establishing that designation requires showing that local compliance changes the identity, behavior or fate of the cells occupying it. This distinction moves the field beyond static mechanical mapping toward a more functional question: whether spatially restricted mechanical environments preferentially support the cells that drive invasion, recurrence and treatment resistance.

The cancer cells associated with these compliant environments may themselves be mechanically distinct. Nanomechanical analysis of patient-derived samples showed that metastatic cancer cells are softer and more mechanically variable than benign cells.^8^ Cellular softness is also associated with increased stemness and tumorigenicity within heterogeneous cancer-cell populations.^9^ A soft cell may deform more readily through confined spaces and adapt to the mechanical stresses encountered during invasion and dissemination. Environmental compliance and cellular softness should not be treated as interchangeable, although they may become coupled during tumor progression. A compliant niche may select or induce softer cells with EMT-associated and stem-like properties. This relationship is not deterministic, however, because cancer cells exposed to the same ECM stiffness can retain intrinsically different mechanical states.^10^ Nor is cellular softness necessarily a fixed lineage property. Once established, a soft malignant state may be persist through cell-intrinsic programs as cells move between mechanical environments or encounter therapeutic pressure. The mechanics of an individual cancer cell may therefore reflect both its current surroundings and its previous mechanical exposures, allowing cellular and environmental mechanics to become spatially and temporally decoupled. Together, these observations point to a multiscale organization of tumor mechanics. ECM mechanics shape the local environments in which malignant states emerge and persist, while cellular mechanics influence how individual cells invade, disseminate and respond to therapeutic attack. Because these mechanical levels are only partially and dynamically coupled, neither bulk matrix measurements nor cell-centered assays alone can fully explain tumor progression or treatment resistance. Consequently, bulk-tissue or ECM stiffness alone cannot identify the cells most likely to invade or resist immune killing. Matrix-normalizing therapies may remodel the tumor environment without eliminating mechanically resistant populations, highlighting the need to address both niche mechanics and cell-intrinsic mechanical states.

Cellular softness becomes particularly consequential during immune attack. Cytotoxic T lymphocytes exert forces on target cells that promote perforin pore formation and target-cell killing,^11^ making the immune synapse a mechanically active interface. Mechanically active LFA-1 integrins further define sites of lytic-granule secretion, coordinating force generation with the focused delivery of perforin and granzyme.^12^ Target-cell mechanics can therefore influence whether immune recognition is effectively converted into cytolysis. Cytotoxic lymphocytes exploit characteristic biophysical vulnerabilities in malignant cells,^13^ whereas soft tumor-repopulating cells resist killing by limiting perforin activity at the immune synapse.^14^ Conversely, increasing cancer-cell stiffness through cholesterol depletion enhances adoptive T-cell therapy.^15^ Together, these studies show that cellular softness can directly contribute to immune resistance. Soft, stem-like and immune-resistant cancer cells may be enriched within compliant niches, but this association is likely to be spatially restricted, dynamically regulated and potentially reversible. Consequently, its biological significance cannot be resolved through bulk mechanical averages or endpoint measurements alone. Instead, it is necessary to connect a cell's prior mechanical experience, or “mechano-memory” with its subsequent molecular identity and therapeutic response.

Resolving this connection requires methods that preserve a durable record of transient mechanical experience at single-cell resolution. Bulk measurements reveal tissue heterogeneity but cannot determine which cells experienced a compliant environment, whereas single-cell assays typically measure current mechanical properties after cells have been removed from their native context. Molecular profiling may also miss mechanically defined states that are rare, transient or reversible. A genetically encoded Mechano-Recorder addresses this limitation by converting softness-associated signaling into durable cellular memory, allowing mechanically experienced cancer cells to be identified in living tumors.^10^ Cells marked by the recorder displayed stem-like properties and pronounced resistance to CAR-T-cell killing, linking a transient softness-associated signal to a persistent malignant and treatment-resistant phenotype.^10^ Recording also makes it possible to isolate these cells after the original mechanical state has changed and examine their spatial distribution, molecular programs and treatment response. Mechanically defined states can thus be studied as cellular histories rather than inferred from a static measurement.

The same mechanical signal can be redirected therapeutically. In our recent study, softness-associated signaling was rewired to induce antigen expression, converting mechanically resistant cancer cells into preferential targets for CAR-T cells.^10^ This strategy defines mechano-reprogramming: the conversion of a disease-associated mechanical input into a therapeutic output. Unlike matrix normalization or global cancer-cell stiffening, mechano-reprogramming does not necessarily attempt to eliminate or reverse the mechanical state itself. Instead, a synthetic circuit senses that state and activates a therapeutic response within the affected cells, thereby converting a signal associated with immune escape into a selective vulnerability. This framework creates opportunities beyond antigen expression. Mechanical inputs could be coupled to pro-apoptotic programs, cytokine release or the recruitment of immune effectors, and they could be combined with tumor-restricted molecular signals to improve specificity. Recording systems may reveal when mechanically resistant states arise during tumor growth, treatment and relapse, while mechano-responsive therapeutic circuits could enable selective intervention as these states emerge.

More broadly, mechano-reprogramming introduces a new therapeutic direction for cancer mechanobiology. Many existing strategies attempt either to normalize the mechanical microenvironment or to alter the physical properties of cancer cells. Mechano-reprogramming instead treats an aberrant mechanical signal as information: a disease-associated state can be sensed, recorded and converted into a therapeutic response. Future designs could integrate mechanical inputs with tumor-specific molecular signals, discriminate between transient and sustained mechanical states, and activate different outputs according to the location or history of the responding cell. Such circuits would not require mechanical heterogeneity to be eliminated; they would use that heterogeneity to identify the cells most likely to survive treatment. By directing therapeutic activity toward these resistant populations, mechano-reprogramming may also expand the utility of treatments that currently fail because they cannot eliminate rare, plastic or immune-evasive cancer cells. Together, this perspective recasts the role of tumor mechanics from a barrier to therapy toward a source for therapeutic selectivity. By translating the mechanical signatures of treatment escape into targeted outputs, mechano-reprogramming may turn mechanical resistance into triggers for therapeutic intervention.

## CRediT authorship contribution statement

**Yunjia Qu:** Conceptualization, Writing – original draft, Writing – review & editing. **Yingxiao Wang:** Conceptualization, Writing – review & editing.

## Ethical approval

This study does not contain any studies with human or animal subjects performed by any of the authors.

## Declaration of competing interests

The authors declare the following financial interests/personal relationships which may be considered as potential competing interests: Yingxiao Wang reports financial support was provided by National Institute of Biomedical Imaging and Bioengineering. Yingxiao Wang reports financial support was provided by National Institute of General Medical Sciences. Yingxiao Wang reports financial support was provided by National Heart Lung and Blood Institute. Yingxiao Wang reports financial support was provided by National Institute of Child Health and Human Development. Yingxiao Wang reports financial support was provided by National Cancer Institute. If there are other authors, they declare that they have no known competing financial interests or personal relationships that could have appeared to influence the work reported in this paper.
